# The Effect of Dietary Approaches to Stop Hypertension (DASH) Diet on Liver Enzyme Level in Adults: A GRADE‐Assessed Systematic Review and Meta‐Analysis of Randomized Clinical Trials

**DOI:** 10.1002/fsn3.71067

**Published:** 2025-11-09

**Authors:** Abbas Mohtashamian, Yasaman Aali, Faezeh Nematolahi, Armin Ebrahimzadeh, Gordon A. Ferns, Majid Ghayour‐Mobarhan

**Affiliations:** ^1^ Department of Nutritional Sciences, Faculty of Medicine Mashhad University of Medical Sciences Mashhad Iran; ^2^ Student Research Committee Mashhad University of Medical Sciences Mashhad Iran; ^3^ Student Research Committee Kashan University of Medical Sciences Kashan Iran; ^4^ Research Center for Biochemistry and Nutrition in Metabolic Diseases Kashan University of Medical Sciences Kashan Iran; ^5^ Nutrition Research Center, School of Nutrition and Food Sciences Shiraz University of Medical Sciences Shiraz Iran; ^6^ Division of Medical Education Brighton and Sussex Medical School Brighton UK; ^7^ Metabolic Syndrome Research Center Mashhad University of Medical Sciences Mashhad Iran; ^8^ International UNESCO Center for Health‐Related Basic Sciences and Human Nutrition Mashhad University of Medical Sciences Mashhad Iran

**Keywords:** ALT, AST, dash diet, liver enzymes, meta‐analysis

## Abstract

Several research studies have shown the beneficial impact of the DASH diet on liver enzyme levels. Nevertheless, the outcomes have been conflicting. Our goal is to offer a summary of the existing documents related to how the dash diet impacts liver enzymes in adults when compared to other dietary patterns. The variables of interest were liver enzymes (ALT, AST). Involved calculating the Weighted Mean Difference (WMD) and 95% confidence intervals (CIs) to assess the effect size. This systematic review and meta‐analysis included 6 studies. Consuming the DASH diet led to a notable alteration in AST levels (mean difference (MD): −3.305 IU/L, 95% CI: −4.709, −1.901, *p* < 0.001). The subgroup analysis revealed that DASH diet consumption could significantly reduce ALT in patients below 44 years old, baseline serum ALT above 30 IU/L, BMI below 30 kg/m^2^, and patients with NAFLD. In addition, DASH diet consumption significantly reduces AST in patients below 44 years old, 8 weeks' duration of study, baseline serum AST above 28 IU/L, BMI below 30 kg/m^2^, and patients with NAFLD. Compared to other dietary patterns, the DASH diet could decrease ALT and AST in general or in subgroup analyses. Additional research is required to gain a better understanding of how the DASH diet affects liver enzymes in adults.

**Trial Registration:** PROSPERO registration no.: CRD42024588334

## Graphical abstract

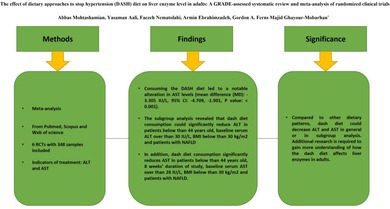

## 1 Introduction

Clinical recommendations and guidelines should include following healthy eating patterns, which are crucial factors in preventing non‐communicable diseases in the world (Benziger et al. 2016). In the past 20 years, the Dietary Approaches to Stop Hypertension (DASH) as guidelines for eating healthily has become a standard. Diet recommendations include consuming low‐fat dairy products, fruits and veggies, whole grains, and limiting intake of sweets, sodium, and red and processed meats (Flynn et al. 2017). The DASH diet is successful in preventing chronic conditions like high blood pressure, heart disease, and type 2 diabetes (DM2) (Forman et al. 2009; Liese et al. 2009; Toledo et al. 2010). Following the DASH diet may improve inflammation and liver function in obese individuals with non‐alcoholic fatty liver disease (NAFLD) (Rooholahzadegan et al. 2023).

NAFLD is related to high enzyme levels and fat accumulation in the hepatic area (Suri et al. 2020). Elevated levels of liver enzymes such as aspartate aminotransferase (AST) and alanine aminotransferase (ALT) in the blood can suggest liver damage (Anderson et al. 1987). The advancement of NAFLD is attributed to inflammation, as findings from recent research suggest (Ucar et al. 2013). A diet rich in pro‐inflammatory components can worsen NAFLD by causing oxidative stress and generating reactive oxygen species (Tyrovolas et al. 2019; Ucar et al. 2013). The DASH diet with high fiber and antioxidant ingredients may be advantageous for reducing insulin resistance and inflammatory conditions (Azadbakht et al. 2007, 2005).

In a controlled randomized clinical trial with double‐blind conditions, obese patients in the DASH group showed a notable decrease in levels of AST and ALT after 8 weeks, when accounting for initial values and weight changes (Rooholahzadegan et al. 2023). But several trials suggested that the DASH diet did not lead to a significant reduction in ALT levels when compared to the control group (Azadbakht et al. 2011; Badali et al. 2023). The DASH diet directed to a more significant reduction in AST and ALT levels in DM2 patients compared to the control diet (Azadbakht et al. 2011). The DASH diet's effect on satiety, weight, waist circumference (WC), and glucose and lipid profile seems to be due to its high dietary fiber content, and these beneficial changes result in decreased liver enzyme levels and a decrease in steatosis for patients with NAFLD (Badali et al. 2023). However, the inconsistency in results—particularly regarding ALT levels—highlights a knowledge gap. Therefore, this research is the initial systematic review and meta‐analysis focusing on examining the impact of the DASH diet on the levels of liver enzymes (ALT and AST) in adults.

## 2 Methods

The present research followed the preferred reporting items of systematic reviews and meta‐analysis (PRISMA) guidelines. This study was registered at PROSPERO (http://www.crd.york.ac.uk/PROSPERO) (Moher et al. 2009).

### 2.1 Search Strategy

To identify relevant research on the impact of the DASH diet on liver enzyme changes until December 1, 2024. We systematically searched electronic databases, for example, Scopus, PubMed/Medline, ISI Web of Science, Embase, and Cochrane databases, by means of MeSH terms and non‐MeSH terms using the following keywords (details of the search strategy in databases are shown in Appendix S1):

(“DASH”[Title/Abstract] OR “dietary approaches to stop hypertension”[MeSH Terms] OR “dietary approaches to stop hypertension”[Title/Abstract]) AND (“Liver”[Title/Abstract] OR “Liver”[MeSH Terms] OR “liver enzyme”[Title/Abstract] OR “Transaminases”[Title/Abstract] OR “Transaminases”[MeSH Terms] OR “Aminotransferase”[Title/Abstract] OR “transpeptidase”[Title/Abstract] OR “Alanine Transaminase”[MeSH Terms] OR “Alanine Transaminase”[Title/Abstract] OR “Alanine Aminotransferase”[Title/Abstract] OR “ALT”[Title/Abstract] OR “SGPT”[Title/Abstract] OR “Aspartate Aminotransferases”[MeSH Terms] OR “Aspartate Aminotransferases”[Title/Abstract] OR “AST”[Title/Abstract] OR “SGOT”[Title/Abstract] OR “Alkaline phosphatase”[Title/Abstract] OR “Alkaline phosphatase”[MeSH Terms] OR “ALP”[Title/Abstract] OR “Gamma‐Glutamyl transferase”[Title/Abstract] OR “GGT”[Title/Abstract] OR “lactate dehydrogenase”[Title/Abstract] OR “L‐Lactate Dehydrogenase”[MeSH Terms] OR “L‐Lactate Dehydrogenase”[Title/Abstract] OR “Dehydrogenase L‐Lactate”[Title/Abstract] OR “Dehydrogenase Lactate”[Title/Abstract] OR “LDH”[Title/Abstract] OR “ast to alt ratio”[Title/Abstract] OR “ast to alt ratio”[Title/Abstract] OR “liver enzyme abnormality”[Title/Abstract] OR “liver enzyme activity”[Title/Abstract] OR “liver function tests”[MeSH Terms] OR “liver function tests”[Title/Abstract] OR “LEA”[Title/Abstract] OR “AST/ALT”[Title/Abstract]) AND (“Randomized Controlled Trial”[Title/Abstract] OR “clinical trial”[Title/Abstract] OR “controlled trial”[Title/Abstract] OR “intervention”[Title/Abstract] OR “Randomized”[Title/Abstract] OR “Randomized”[Title/Abstract] OR “randomly”[Title/Abstract] OR “placebo”[Title/Abstract] OR “trial”[Title/Abstract] OR “assignment”[Title/Abstract] OR “RCT”[Title/Abstract] OR “cross‐over”[Title/Abstract] OR “parallel”[Title/Abstract] OR “single‐blind”[Title/Abstract] OR “double‐blind”[Title/Abstract] OR “Controlled Clinical Trial”[Title/Abstract]).

### 2.2 Study Selection

The screening of articles was carried out independently through two authors (Y.A. and A.M.) based on title and abstract. We calculated the kappa statistic to determine the level of agreement between reviewers for study selection using SPSS software (ver. 26). To this end, the following interpretation of kappa was used: chance agreement (≤ 0), slight agreement (0.01–0.20), fair agreement (0.21–0.40), moderate agreement (0.41–0.60), substantial agreement (0.61–0.80), almost perfect agreement (0.81–0.99). In this stage, there was perfect agreement in study selection between the reviewers (*К* statistic, 0.84; *p* < 0.001). The studies included in the analysis were selected based on the PICOS criteria (participants, interventions, comparisons, outcomes, and study design), shown in Table 6. Studies were pooled along with the following criteria: (1) studies performed in adults (≥ 18 years), (2) RCT (crossover or parallel), (3) evaluation of the DASH diet on liver enzymes, (4) report means and standard deviations (SDs). The search was narrow to human studies and did not have any language restrictions. Exclusion criteria included: (1) animal and review studies, (2) non‐RCT or RCT without a placebo group, (3) studies conducted on children or adolescents, case reports, conference papers, dissertations, editorial papers, and books.

### 2.3 Data Extraction

Two assessors (A.M. and F.N.) performed an unaffiliated evaluation of the RCTs. Ineligible studies were excluded. Finally, К statistic was calculated to determine the agreement level between reviewers for data extraction using SPSS software (ver. 26). In this stage, there was perfect agreement in study selection between the reviewers (*К* statistic, 0.87; *p* < 0.001). Data extracted included the following: publication details (first author's full name, year of publication, and country where the study was conducted), characteristics of participants (age, health status, body mass index, and gender), characteristics of the study (number of participants, type of control treatment, duration of intervention, study design), and outcomes (ALT, AST). The lack of this information, the mean value at the initiation was deducted from the mean value at the end of the intervention, and the mean changes were computed.

### 2.4 Quality Assessment

Two researchers (F.N. and A.M.) evaluated the quality of the studies included using the Cochrane Collaboration tool separately (Higgins et al. 2011). Among the six included RCTs, it was found that all studies had a low risk of bias (Azadbakht et al. 2011; Badali et al. 2023; Panizza et al. 2019; Razavi Zade et al. 2016; Sangouni, Nadjarzadeh, et al. 2024; Sangouni, Hosseinzadeh, and Parastouei 2024). All studies reported randomization and described the randomization procedure (Azadbakht et al. 2011; Badali et al. 2023; Panizza et al. 2019; Razavi Zade et al. 2016; Sangouni, Nadjarzadeh, et al. 2024; Sangouni, Hosseinzadeh, and Parastouei 2024). Details of the quality assessment results are shown in Table 1. Also, the *К* statistic was calculated to determine the level of agreement between reviewers for assessing the quality of included studies using SPSS software (ver. 26). Additionally, the general quality of the evidence related to liver enzyme levels was evaluated using the GRADE evidence profiles, which indicate that the overall quality of evidence for ALT and AST levels is low and high, respectively (Table 2).

**TABLE 1:** Quality assessment of the studies included using the Cochrane Collaboration tool investigating the effect of the DASH diet on liver enzyme levels.

Studies	Random sequence generation	Allocation concealment	Blinding of participants and personnel	Blinding of outcome assessment	Incomplete outcome data	Selective outcome reporting	Other potential threats to validity	General risk of bias
Azadbakht et al. (2011)	L	U	H	U	L	L	L	Low
Panizza et al. (2019)	L	L	H	U	L	L	L	Low
Sangouni, Nadjarzadeh, et al. (2024)	L	L	H	U	L	L	L	Low
Badali et al. (2023)	L	L	L	H	L	L	L	Low
Sangouni, Hosseinzadeh, and Parastouei (2024)	L	L	H	U	L	L	L	Low
Razavi Zade et al. (2016)	L	L	H	U	L	L	L	Low

Abbreviations: H, high risk of bias; L, low risk of bias; U, unclear risk of bias.

**TABLE 2:** Grade profile regarding the effect of DASH diet on liver enzyme levels.

Quality assessment						Quality of evidence
Outcomes	Risk of bias	Inconsistency	Indirectness	Imprecision	Publication bias	
ALT	No serious limitation	Very serious limitation	No serious limitation	Serious limitation	No serious limitation	Low ⊕⊕◯◯
AST	No serious limitation	No serious limitation	No serious limitation	No serious limitation	No serious limitation	High ⊕⊕⊕⊕

Abbreviations: ALT, alanine aminotransferase; AST, aspartate aminotransferase.

### 2.5 Statistical Analysis

In the present study, mean and SD changes were used to define the overall effect size. SD changes were calculated based on the formula: (SD_baseline_^2^ + SD_final_^2^) − (2 × *R* × SD_baseline_ × SD_final_) assuming a correlation coefficient of 0.5, as a conservative estimate for R which ranges between 0 and 1. If SE was used in the studies, the formula (SD = SE × √*n* (*n* = the number of individuals in each group)) was used to convert standard errors (SE), interquartile ranges (IQRs), and 95% confidence interval (CI) to SD (Hozo et al. 2005).

## 3 Results

### 3.1 Study Selection

**FIGURE 1:**
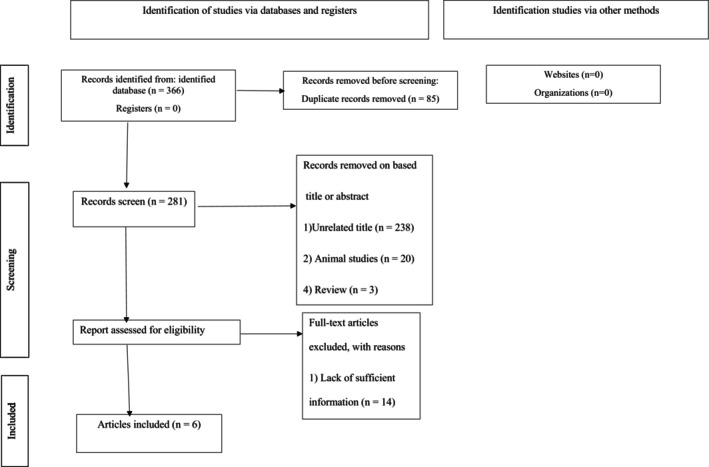
Flow diagram of the study selection procedure showing the number of eligible studies for the meta‐analysis of the effect of DASH *diet* on liver enzymes.

Two authors independently screened the title, abstract, and full text of the articles. In this stage, there was perfect agreement in study screening between the reviewers (*К* statistic, 0.86; *p* < 0.001). Eighty‐five duplicate articles were removed from a total of 366 articles found in PubMed‐MEDLINE, Cochrane Library, Scopus, Web of Science, Google Scholar databases, and Embase. An additional 261 articles were eliminated after reviewing their titles and abstracts. After reading all 20 articles, we excluded 14 studies for not having enough information (Figure 1).

### 3.2 Study Characteristics

A summary of the features of qualifying studies can be found in Table 3. The articles that were included had a sample size ranging from 40 to 67 participants. Among the 6 studies analyzed, 5 were conducted in Iran and 1 was conducted in the USA. The trials lasted for a period ranging from 8 to 12 weeks. Research has been carried out on both males and females in all studies. There were five studies that used a parallel design and one study that used a crossover design. Participant characteristics were different in various studies, with a focus on special and diseased populations like metabolic syndrome, T2DM, NAFLD, and healthy.

**TABLE 3:** Demographic characteristics of the included studies.

Author (year)	Country	Subjects	Mean age	Mean of BMI at the baseline	Design	Intervention group	Comparator group	Duration (weeks)	Participants	Outcomes
Sangouni, Nadjarzadeh, et al. (2024)	Iran	I: 30 C: 29	44.43	30.49	Parallel—A randomized controlled trial	Dash diet	Healthy diet (50%–55% carb, 15%–20% pro, and 30% fat)	12	Metabolic syndrome	ALT, AST
Sangouni, Hosseinzadeh, and Parastouei (2024)	Iran	I: 34 C: 33	43.14	29.72	Parallel—A randomized controlled trial	Dash diet	Healthy diet (50%–55% carb, 15%–20% pro, and 30% fat)	12	Obese adults with NAFLD	ALT, AST
Badali et al. (2023)	Iran	I: 20 C: 20	38.8	33.43	Parallel—A randomized controlled trial	Dash diet	LCD diet	8	Obese adults with NAFLD	ALT, AST
Panizza et al. (2019)	USA	I: 30 C: 30	46.2	30.8	Parallel—A randomized controlled trial	Dash diet	Intermittent energy restriction combined with a Mediterranean diet (IER + MED)	12	Healthy adults	ALT, AST
Razavi Zade et al. (2016)	Iran	I: 30 C: 30	39.7	27.2	Parallel—A randomized controlled trial	Dash diet	Healthy diet (50%–55% carb, 15%–20% pro, and 30% fat)	8	Obese adults with NAFLD	ALT, AST
Azadbakht et al. (2011)	Iran	I: 62 C: 62	55	—	Crossover—A randomized controlled trial	Dash diet	Healthy diet (50%–55% carb, 15%–20% pro, and 30% fat)	8	T2DM	ALT, AST

### 3.3 Meta‐Analysis Results

**FIGURE 2:**
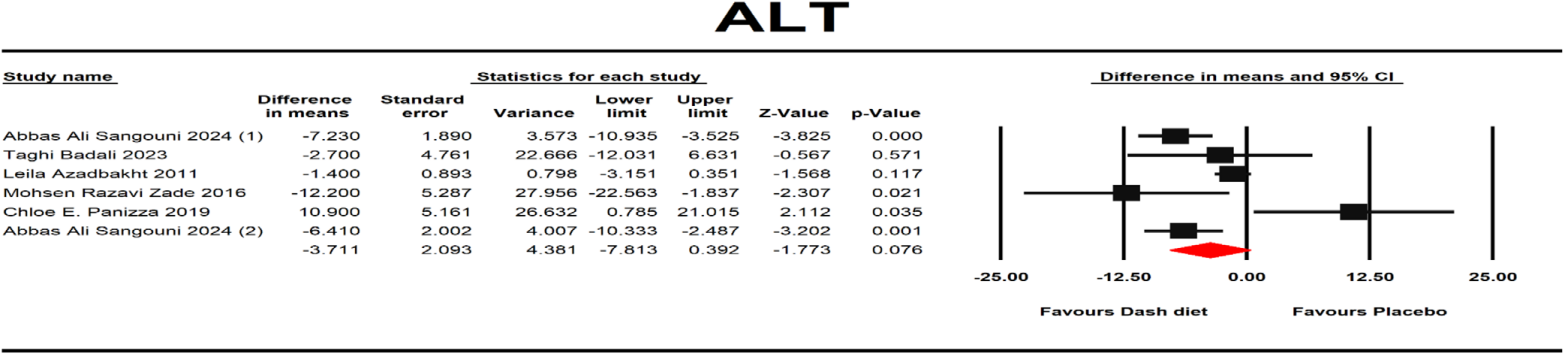
Forest plot of randomized controlled trials that compares the effects of the intervention (DASH diet) and the control (placebo) groups on ALT. Effects are presented as means and standard error (SE) for within‐group change from baseline and standardized (Std) mean differences with random 95% CI. The findings exhibited no significant alteration in ALT levels after consuming the DASH diet (mean difference (MD): −3.71 IU/L, 95% CI: −7.813, 0.392, *p* = 0.076).

**FIGURE 3:**
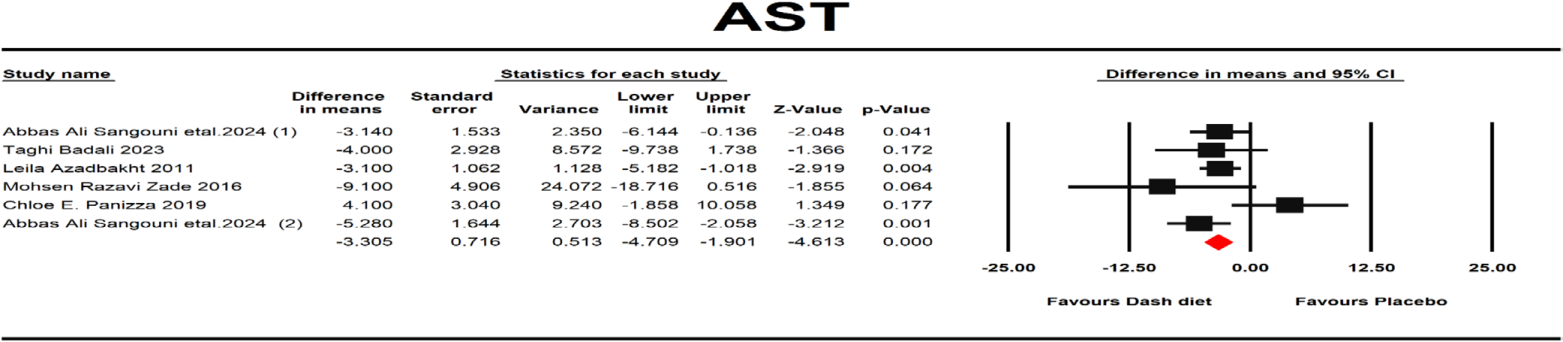
Forest plot of randomized controlled trials that compares the effects of the intervention (DASH diet) and the control (Placebo) groups on AST. Effects are presented as means and standard error (SE) for within‐group change from baseline and standardized (Std) mean differences with random 95% CI. The results showed that consuming the DASH diet led to a notable AST change (mean difference (MD): −3.305 IU/L, 95% CI: −4.709, −1.901, *p* < 0.001).

ALT was reported as an outcome measure in six studies with a total of 348 contributors. The pooled findings from the random effects model exhibited no significant alteration in ALT levels after consuming the DASH diet (mean difference (MD): −3.71 IU/L, 95% CI: −7.813, 0.392, *p* = 0.076) (Figure 2) with substantial heterogeneity among the studies (*I* ^2^ = 76.62%, *p* = 0.001, Mean PI = −3.71, 95% PI = −16.37, 8.95, TAU^2^ = 16.43). Also, one‐stage dose response, Galbraith plot, and Hartung‐Knapp adjustment analysis for the ALT outcome was done (95% CI: −8.15, 7.37, *p* = 0.9). AST was reported as an outcome measure in six studies with a total of 348 participants. The fixed‐effects model exhibited that consuming the DASH diet led to a notable AST change (mean difference (MD): −3.305 IU/L, 95% CI: −4.709, −1.901, *p* < 0.001) (Figure 3). There was no notable heterogeneity between these studies (*I* ^2^ = 43.67%, *p* = 0.114, Mean PI = −3.3, 95% PI = −8.37, 1.77, TAU^2^ = 2.83). Also, one‐stage dose response, Galbraith plot, and Hartung‐Knapp adjustment analysis for the AST outcome was done (95% CI: −4.54, 3.76, *p* = 0.81).

### 3.4 Sensitivity Analysis

**FIGURE 4:**
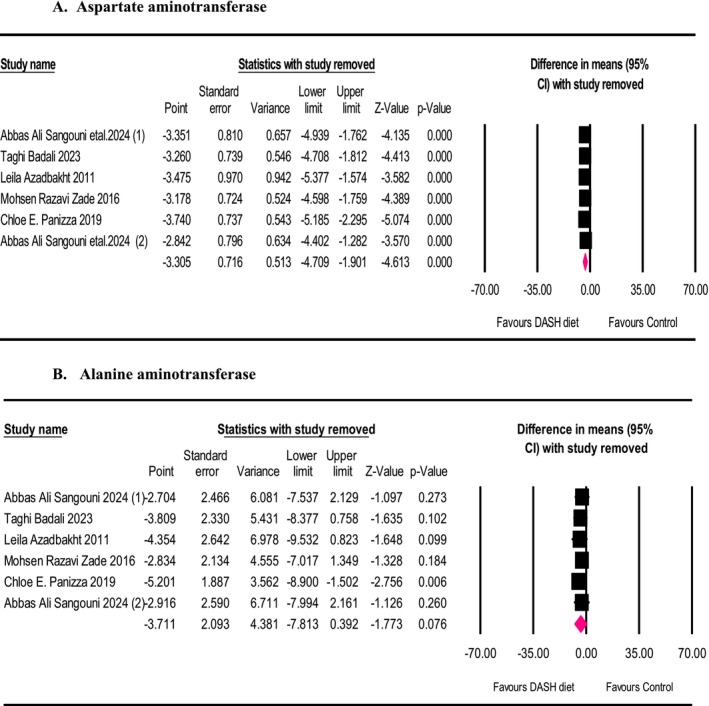
Forest plot of the leave‐one‐out sensitivity analysis of the effects of the intervention (DASH diet) and the control (placebo) groups on AST (A) and ALT (B). Effects are presented as means and standard error (SE) for within‐group change from baseline and standardized (Std) mean differences with random 95% CI.

We conducted a leave‐one‐out sensitivity analysis to detect each trial's effect on the pooled effect size. The effect size for the influence of the DASH diet on AST levels was robust in the leave‐one‐out sensitivity analysis, suggesting that omitting each single trial did not significantly affect the meta‐analysis results (Figure 4 panel A). However, the effect size aimed at the impact from the DASH diet on ALT levels was sensitive to the study of Panizza et al. (2019). Eliminating this study from the analysis significantly changed the meta‐analysis results (Figure 4 panel B).

### 3.5 Results From Subgroup Analysis

The results of the subgroup analyses are provided in Table 4. We stratified the studies based on the type of study population, age (years), duration (weeks), BMI, and baseline serum ALT and AST. The subgroup analysis presented that DASH diet consumption could significantly reduce ALT in patients below 44 years old (WMD: −6.542 IU/L, 95% CI: −9.957, −3.127, *p* < 0.001), baseline serum ALT over 30 IU/L (WMD: −7.182 IU/L, 95% CI: −9.79, −4.575, *p* < 0.001), BMI below 30 kg/m^2^ (WMD: −7.237 IU/L, 95% CI: −11.207, −3.266, *p* < 0.001), and patients with NAFLD (WMD: −6.542 IU/L, 95% CI: −9.957, −3.127, *p* < 0.001). In addition, DASH diet consumption significantly reduces AST in patients below 44 years old (WMD: −5.298 IU/L, 95% CI: −7.994, −2.601, *p* < 0.001), 8 weeks' duration of study (WMD: −3.439 IU/L, 95% CI: −5.357, −1.521, *p* < 0.001), baseline serum AST over 28 IU/L (WMD: −4.382 IU/L, 95% CI: −6.524, −2.239, *p* < 0.001), BMI below 30 kg/m^2^ (WMD: −5.666 IU/L, 95% CI: −8.721, −2.61, *p* < 0.001), and patients with NAFLD (WMD: −5.298 IU/L, 95% CI: −7.994, −2.601, *p* < 0.001).

**TABLE 4:** Results of subgroup analysis of the included trials regarding the effects of DASH diet on serum liver enzymes.

	Number of comparison	WMD	95% CI	*p*	*I* ^2^ (%)	P‐Heterogeneity	95% PI
Subgroup analyses for ALT outcome							
Age (years)							
≤ 44	3	−6.54	−9.95, −3.12	< 0.001	0	0.406	−62.61, 49.53
> 44	3	−1.08	−7.36, 5.19	0.735	86.03	0.001	−66.69, 64.53
Duration (weeks)							
8	3	−3.85	−9.5, 1.8	0.182	51.21	0.129	−67.04, 59.34
12	3	−2.79	−9.75, 4.16	0.431	82.02	0.004	−71.21, 65.63
Baseline serum ALT (IU/L)							
≤ 30	3	1.21	−5.57, 8.01	0.725	64.49	0.06	−66.58, 69
> 30	3	−7.18	−9.79, −4.57	< 0.001	0	0.592	−61.39, 47.03
BMI (kg/m^2^)							
≤ 30	2	−7.23	−11.2, −3.26	< 0.001	4.65	0.306	−64.8, 50.34
> 30	3	−0.44	−10.75, 9.89	0.932	81.98	0.004	−84.92, 84.04
Population							
NAFLD	3	−6.54	−9.95, −3.12	< 0.001	0	0.406	−62.61, 49.53
Other diseases	3	−1.08	−7.36, 5.19	0.735	86.03	0.001	−56.9, 59.06
Subgroup analyses for AST outcome							
Age (years)							
≤ 44	3	−5.298	−7.99, −2.6	< 0.001	0	0.671	−32.87, 22.29
> 44	3	−1.805	−4.89, 1.28	0.251	61.55	0.074	−31.05, 27.45
Duration (weeks)							
8	3	−3.43	−5.35, −1.52	< 0.001	0	0.48	−28.13, 21.27
12	3	−2.16	−6.41, 2.09	0.32	72.85	0.025	−37.03, 32.71
Baseline serum AST (IU/L)							
≤ 28	3	−1.48	−5.69, 2.73	0.491	62.23	0.071	−36.14, 33.18
> 28	3	−4.38	−6.52, −2.23	< 0.001	0	0.391	−29.89, 21.13
BMI (kg/m^2^)							
≤ 30	2	5.66	−8.72, −2.61	< 0.001	0	0.46	−34.77, 23.45
> 30	3	−1.39	−5.8, 3.009	0.535	60.32	0.08	−36.98, 34.2
Population							
NAFLD	3	−5.29	−7.99, −2.6	< 0.001	0	0.671	−32.87, 22.29
Other diseases	3	−1.8	−4.89, 1.28	0.251	61.55	0.074	−31.05, 27.45

### 3.6 Publication Bias

**FIGURE 5:**
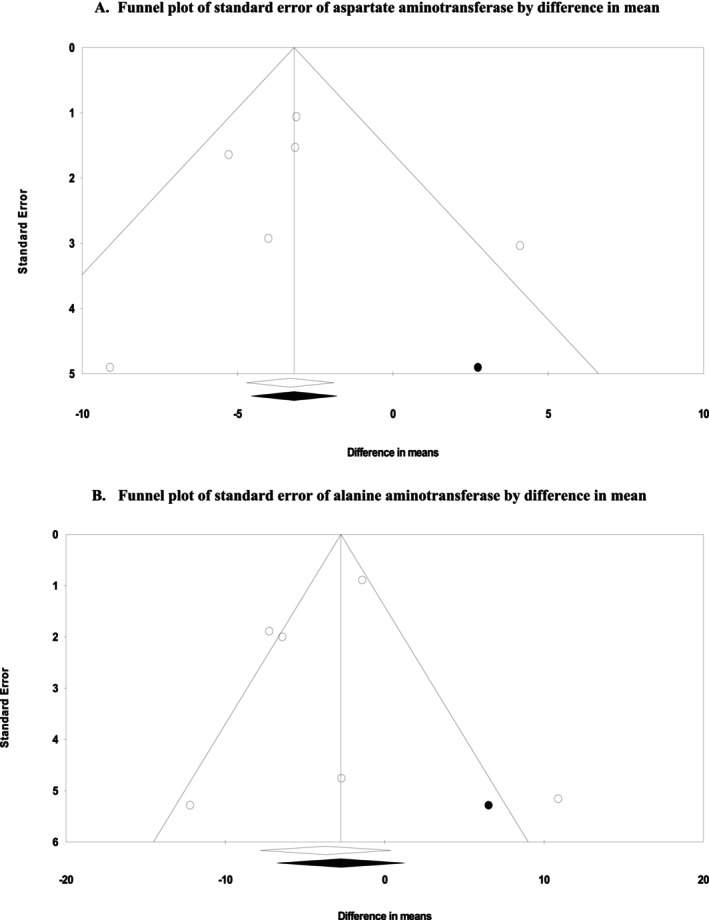
Funnel plots for publication bias assessment of the intervention (DASH diet) and the control (placebo) groups on AST (A) and ALT (B). Effects are presented as means and standard error (SE) for within‐group change from baseline. After applying the “trim and fill” method, one potentially missing study was imputed for the meta‐analysis of each AST or ALT for adjusting publication bias.

The funnel plots of the influence of the DASH diet on ALT and AST levels are shown in Figure 5. After applying the “trim and fill” method, one potentially missing study was imputed for the meta‐analysis of each AST or ALT for adjusting publication bias (Figure 5, Appendix S2). Table 5 summarizes the results of Begg's rank correlation, Egger's linear regression, “fail‐safe *N*” tests, and correlated effect size (Table 6, Figure 1).

**TABLE 5:** Publication bias for ALT and AST.

	Corrected effect size		Begg's rank correlation test			Egger's liner regression test					Fail‐safe *N* test
	WMD	95% CI	Kendall's Tau	*z*	*p*	Intercept	95% CI	*t*	df	*p*	*n*
ALT	−2.75	−6.76, 1.25	0	< 0.001	1	−0.67	−5.06, 3.70	0.42	4	0.68	17
AST	−3.17	−4.56, −1.78	−0.13	0.37	0.70	0.10	−3.76, 3.96	0.07	4	0.94	21

**TABLE 6:** PICOS criteria used to define the research question.

Participants	Interventions	Comparisons	Outcomes	Study design
All humans	Dash Diet	Liver enzymes	ALT and AST	PR and CR

## 4 Discussion

Based on current understanding, this research represents the first comprehensive analysis and meta‐analysis evaluating the influence of the DASH diet on liver enzyme levels in adults. It includes findings from six RCTs (Azadbakht et al. 2007; Badali et al. 2023; Panizza et al. 2019; Razavi Zade et al. 2016; Sangouni, Nadjarzadeh, et al. 2024; Sangouni, Hosseinzadeh, and Parastouei 2024). The meta‐analysis reveals that adherence to the DASH diet leads to significant reductions in levels of AST, while the impact on ALT was not statistically significant.

During subgroup analysis, a notable decrease in ALT was observed in participants under 44 years of age, with a baseline serum ALT over 30 IU/L, a BMI below 30 kg/m^2^, and patients with NAFLD. Furthermore, the Dash diet eating plan significantly decreases AST levels in patients younger than 44 years old, with a study duration of 8 weeks, a baseline serum AST over 28 IU/L, a BMI below 30 kg/m^2^, and patients with NAFLD.

In the current study, there was no significant heterogeneity regarding AST levels among the included studies, which makes our results about AST levels more reliable. However, interpreting our findings accurately for ALT levels is more challenging due to the significant heterogeneity regarding ALT levels among the included studies. This apparent heterogeneity suggests that Dash diet consumption may have varying impacts on ALT levels across different populations. Notably, the randomized controlled trials included in the current meta‐analysis involved adult participants of both genders, including healthy individuals, as well as patients with metabolic syndrome, T2DM, or NAFLD (Azadbakht et al. 2011; Badali et al. 2023; Panizza et al. 2019; Razavi Zade et al. 2016; Sangouni, Nadjarzadeh, et al. 2024; Sangouni, Hosseinzadeh, and Parastouei 2024).

Randomized controlled trials examining the impact of the DASH diet on AST and ALT levels have typically shown conflicting results (Azadbakht et al. 2011; Badali et al. 2023; Panizza et al. 2019; Razavi Zade et al. 2016; Sangouni, Nadjarzadeh, et al. 2024; Sangouni, Hosseinzadeh, and Parastouei 2024). Similar to our findings, some studies reported no significant reduction in ALT levels (Azadbakht et al. 2011; Badali et al. 2023), though certain studies have shown a notable reduction in ALT levels among participants following the DASH diet, in contrast to those in the control group (Razavi Zade et al. 2016; Sangouni, Nadjarzadeh, et al. 2024; Sangouni, Hosseinzadeh, and Parastouei 2024). Additionally, similar to our findings, certain studies have demonstrated a notable decrease in AST levels when following the DASH diet (Azadbakht et al. 2011; Badali et al. 2023; Sangouni, Nadjarzadeh, et al. 2024; Sangouni, Hosseinzadeh, and Parastouei 2024), while others have not observed a significant divergence compared to the control group (Razavi Zade et al. 2016).

As mentioned, similar to our findings, some trials indicated that the DASH diet did not significantly reduce ALT levels compared to the control group (Azadbakht et al. 2011; Badali et al. 2023). One trial noted that this could be due to the differences in baseline ALT levels between the DASH diet group and the control group (Azadbakht et al. 2011). The findings of the present meta‐analysis also showed that the participants' baseline serum ALT levels, as well as AST levels, were one source of heterogeneity. The results of the sub‐group analysis exhibited that a significant reduction in ALT and AST was observed in participants with baseline serum ALT over 30 IU/L and baseline serum AST over 28 IU/L, respectively. Additionally, in the current study's sub‐group analysis, a significant reduction in ALT and AST levels was observed in patients with NAFLD. This could also be attributed to the higher baseline level of these enzymes in patients with NAFLD. Moreover, in sub‐group analysis, a significant reduction in ALT and AST levels was found in participants under 44 years of age but not in participants over 44 years of age. This finding could again be due to the higher level of ALT typically found in this age group, as investigations have revealed that serum ALT levels are associated with age, tending to increase from the first to the fourth decade of life and decreasing after that (Dufour et al. 2000; Kang et al. 2011). Similarly, some investigations have found that AST serum levels are likewise negatively affected by age (Bussler et al. 2018).

The main reason for the positive effects of the DASH diet is its abundance of vegetables, whole grains, legumes, fruits, and nuts. Additionally, the DASH diet focuses on restricting consumption of saturated fat, processed and red meats, salt, added sugars, and sugar‐sweetened beverages (Harsha et al. 1999). This diet consists of high‐fiber foods with a low glycemic index that are not dense in energy and also help lower cholesterol levels (Chiavaroli et al. 2019; Suri et al. 2020). Earlier studies have revealed that patients with NAFLD who followed the DASH diet experienced notable reductions in weight, BMI, and WC when compared to those on the control diet (Razavi Zade et al. 2016; Yazici et al. 2009). Besides, prior evidence has revealed that consuming vegetables, fruits, and whole grains is contrariwise associated with the severity of fatty liver and levels of liver enzymes (Campbell 2017; Panbehkar‐Jouybari et al. 2021). Various mechanisms have been proposed to explain how the DASH diet may impact liver enzyme levels. Consuming less simple sugar and more fiber, magnesium, and calcium in the DASH diet could result in reduced levels of liver enzymes in the blood (Razavi Zade et al. 2016). The DASH diet's effect on liver enzymes is also due to its various antioxidants and bioactive compounds. One of the most important of these is polyphenols, which are bioactive components found in vegetables and fruits. These elements are able to reduce the synthesis of lipids in the liver, enhance the elimination of fat, and result in enhancements in the factors that contribute to the advancement of fatty liver and levels of liver enzymes (Salomone et al. 2016; Sangouni et al. 2023; Sangouni, Hosseinzadeh, and Parastouei 2024). Studies have shown that a mix of polyphenols is more effective in treating fatty liver compared to just one type of polyphenol supplement (Elgebaly et al. 2017; Mahmoodi et al. 2020). Moreover, there is evidence supporting that the intake of whole grains decreases lipid accumulation in hepatocytes and decreases serum levels of AST and ALT (Schutte et al. 2018), whereas consuming refined grains activates the buildup of TGs in the liver (Dorosti et al. 2020; Fardet 2010; Hoevenaars et al. 2019; Schutte et al. 2018). The DASH diet contains a large quantity of whole grains, fruits, and vegetables that offer a high level of dietary fiber.

Fibers canister increase the number of beneficial bacteria in the gut and improve the performance of gut microbiota (Holscher 2017; Makki et al. 2018). Then, short‐chain fatty acids (SCFAs) (acetate, propionate, and butyrate) derived from fermentable dietary fibers by gut microbiota have the ability to enhance the liver's fat clearance, boost insulin sensitivity, and reduce inflammation pathway activation (Canfora et al. 2015; Den Besten et al. 2015; Mollica et al. 2017). Moreover, there is a connection between obesity and fatty liver. Certain mechanisms listed earlier, through which the DASH diet lessens the intensity of fatty liver, may also lower obesity. Moreover, consuming fiber can also prolong gastric emptying and feelings of fullness, while reducing both hunger and the absorption of macronutrients (Dayib et al. 2020). Nevertheless, it should be noted that some studies have suggested that it's not clear whether the welfares of the DASH eating pattern on liver enzyme levels are influenced by weight loss. But significant associations between DASH eating patterns and liver enzyme levels have stayed even after adjusting for weight, suggesting that the DASH diet has beneficial effects self‐governing of weight reduction (Azadbakht et al. 2011). In this regard, the studies' findings are inconsistent (Azadbakht et al. 2011; Dayib et al. 2020). This may be due to the various populations of the investigations with different baseline weights. More well‐designed studies with larger sample sizes are obligatory to determine whether the beneficial effects of the DASH dietary pattern are dependent on weight reduction or not.

When subgroup analysis was conducted, the effect of the DASH diet on serum ALT levels was not significant in the trials with a duration of 8 or 12 weeks. However, it's important to interpret this result cautiously due to significant heterogeneity in the trials with intervention durations of 8 or 12 weeks. Correspondingly, the DASH diet had a significant effect on serum AST levels in trials with a duration of 8 weeks but not in trials with a duration of 12; yet due to significant heterogeneity in the trials with intervention durations of 12 weeks, this result should be interpreted cautiously.

In the subgroup analysis, participants with a BMI below 30 kg/m^2^ showed a significant decrease in ALT and AST levels. Regardless, because there is heterogeneity in the trials on participants with a BMI above 30 kg/m^2^, the obtained result should be interpreted cautiously. Some studies have shown a correlation between liver enzyme levels and BMI, with levels also varying significantly by gender (Nakamura et al. 1998). However, some others demonstrate a relation between BMI and serum liver enzyme activity, with the liver enzyme activity of obese subjects (BMI greater than 30 kg/m^2^) increasing compared with that of normal subjects in both genders (Salvaggio et al. 1991). Moreover, some trials indicated that the DASH group had greater reductions in BMI compared with the control group (Badali et al. 2023). Considering the findings of our meta‐analysis and the results of previous studies, further research is needed to assess the effect of adherence to the DASH diet on liver enzyme levels in individuals with different BMI levels and genders.

The DASH eating pattern has distinct properties that set it apart from other healthy diets that assist in managing liver diseases and reducing liver enzymes. This diet is recommended for lowering blood pressure and improving cardiovascular risk factors. It seems to have greater beneficial impacts on individuals with increased cardiometabolic risk (Siervo et al. 2015). Meanwhile, a significant number of patients with fatty liver diseases have increased cardiometabolic risk (Aneni et al. 2015; El Hadi et al. 2019; Lim et al. 2015; Stefan et al. 2019). In fact, a higher cardiometabolic index is related to NAFLD risk (Zou et al. 2022). Thus, the DASH eating pattern seems to be a reasonable approach to managing NAFLD and cardiometabolic risk together (Chiavaroli et al. 2019). Furthermore, the evidence suggests that following the DASH diet is associated with improved mental health (Faghih et al. 2020). Another applicable property of the DASH diet is its wide variety of food options, making it comfortable for most of the diet participants to adhere to it (Moore and Jenkins 2011; Windhauser et al. 1999). Simply following the DASH diet plan, even to a small degree, is linked to a decreased likelihood of all‐cause and cause‐specific mortality. Greater adherence to this diet further strengthens the risk‐reducing association (Soltani et al. 2020).

The present study had some strong points. Up until now, no other study has conducted a systematic review and meta‐analysis to evaluate how the DASH dietary plan impacts liver enzyme levels in adults. To cover all relevant literature, a complete search was conducted using PRISMA guidelines (Page et al. 2021) across six databases: Scopus, PubMed, ISI Web of Science, Embase, Cochrane databases, and Google Scholar. Furthermore, the reference lists of the related reviews were searched. This study also utilized a snowball search technique to include other relevant trials that may have been missed. Then, standard methodologies were used to assess heterogeneity, meta‐analysis, sensitivity analysis, and publication bias. Also, the quality of the included studies was assessed according to the Cochrane guideline. All included trials had a low risk of bias, strengthening the inference of a cause‐and‐effect association. Additionally, the GRADE evidence profiles were used to evaluate the overall quality of evidence related to the effect of the DASH diet on liver enzyme levels. Based on the GRADE evidence profiles, no serious limitation was found regarding the risk of bias, indirectness, and publication bias regarding the effect of the DASH dietary plan on ALT and AST levels. In addition, according to the GRADE evidence profiles, the quality of evidence regarding the effect of the DASH dietary plan on AST was high. Thus, we are very confident that the true effect is close to the estimate (Kirmayra et al. 2021). However, the present study has some limitations that need to be taken into account. The included RCTs were limited and had a modest sample size, which resulted in inadequate statistical power to detect a certain effect in the present meta‐analysis. It should be noted that according to the GRADE evidence profiles, the quality of evidence regarding the effect of the DASH dietary plan on ALT was low. A sensitivity analysis was performed to assess the robustness of the results. This involved modifying the inclusion criteria and systematically excluding specific studies to observe how these changes affected the overall findings, thereby providing a clearer understanding of the impact of the DASH dietary plan on ALT. As a result, our confidence in the estimate of this effect is limited (Kirmayra et al. 2021).

It is worth noting that some of the included RCTs did not provide sufficient data on the participants' waist circumference, fat mass, total lean body mass, and physical activity level (Azadbakht et al. 2011; Badali et al. 2023; Sangouni, Nadjarzadeh, et al. 2024; Sangouni, Hosseinzadeh, and Parastouei 2024). Therefore, it was not possible to include them in sub‐group analysis based on these variables, even though these variables could affect the results (Bekkelund and Jorde 2019; Samadi et al. 2007; Stranges et al. 2004; Valizadeh et al. 2011). Moreover, due to the limited available data in the trials, it was not possible to analyze the effect of DASH diet consumption on alkaline phosphatase (ALP) serum levels (Badali et al. 2023; Sangouni, Nadjarzadeh, et al. 2024).

The sensitivity analysis in the current study revealed that removing each trial would not significantly change the meta‐analysis results about the effect size of the DASH diet on AST levels; nevertheless, the effect size for the impact of the DASH diet on ALT levels was sensitive to the study conducted by Panizza et al. (2019). In other words, omitting this investigation from the analysis significantly changes the meta‐analysis results regarding the consequence of the DASH diet on ALT levels; consequently, it is important to interpret our findings with caution.

Furthermore, the included RCTs were heterogeneous in terms of the participants' mean age and BMI, population, intervention duration, and baseline serum ALT and AST levels. It seems that potential differences in the population, participants' mean age, and baseline serum ALT and AST levels may have a confounding impact on the changes in serum ALT and AST levels. Therefore, the interpretation of our findings should be done cautiously. We did a subgroup analysis to find the possible sources of heterogeneity. However, in some cases, these analyses were not able to resolve this problem.

Future research should conduct more carefully planned randomized controlled trials with bigger participant groups and extended intervention periods to investigate the impact of the DASH dietary plan on liver enzyme levels. These trials can mainly focus on the impact of the DASH dietary plan on liver enzyme levels, such as ALP, in patients of different races and different countries with NAFLD. Moreover, it is recommended that these studies supply adequate data on the participants' weight, height, BMI, waist circumference, fat mass, total lean body mass, and physical activity level.

## 5 Conclusions

Compared to other dietary patterns, the dash diet could decrease ALT and AST in general or in subgroup analysis. More research is required to gain a better understanding of how the dash diet impacts liver enzymes in adults.

## Author Contributions

A.M. conceived the study. A.M. and M.G.‐M. wrote the proposal. A.M. carried out the literature search. A.M. and Y.A. carried out the literature screening. A.M. and F.N. carried out data extraction and independent reviewing. A.M. and F.N. accompanied the quality evaluation of included studies. A.M. conducted data analysis and interpretation. A.M., Y.A., and F.N. wrote the manuscript. A.M. and M.G.‐M. conducted the critical revision of the manuscript. All authors read the manuscript and approved it.

## Conflicts of Interest

The authors declare no conflicts of interest.

## Supporting information


**Appendix S1:** Supporting Information.

## Data Availability

The data sets generated or analyzed during the present study are not publicly accessible due to the regulations of the Research Center for Biochemistry and Nutrition in Metabolic Diseases of Mashhad University of Medical Sciences. However, they can be accessed from the corresponding author upon logical request.
