# Supplementary material for: The Effect of Dietary Approaches to Stop Hypertension (DASH) Diet on Liver Enzyme Level in Adults: A GRADE‐Assessed Systematic Review and Meta‐Analysis of Randomized Clinical Trials
Source: Food Sci Nutr. 2025 Nov 9;13(11):e71067. doi: 10.1002/fsn3.71067 (PMC12597769; doi:10.1002/fsn3.71067)
Supplement: Supplementary file 1 — Appendix S1: Supporting Information. [file FSN3-13-e71067-s001.docx]

**Supplemental Information:**

Appendix S1. Details of the search strategy in databases.

**DATE: 1 August 2024**

|  |  |
| --- | --- |
| **Pubmed (27)** | ("DASH"[Title/Abstract] OR "dietary approaches to stop hypertension"[MeSH Terms] OR "dietary approaches to stop hypertension"[Title/Abstract]) AND ("Liver"[Title/Abstract] OR "Liver"[MeSH Terms] OR "liver enzyme"[Title/Abstract] OR "Transaminases"[Title/Abstract] OR "Transaminases"[MeSH Terms] OR "Aminotransferase"[Title/Abstract] OR "transpeptidase"[Title/Abstract] OR "Alanine Transaminase"[MeSH Terms] OR "Alanine Transaminase"[Title/Abstract] OR "Alanine Aminotransferase"[Title/Abstract] OR "ALT"[Title/Abstract] OR "SGPT"[Title/Abstract] OR "Aspartate Aminotransferases"[MeSH Terms] OR "Aspartate Aminotransferases"[Title/Abstract] OR "AST"[Title/Abstract] OR "SGOT"[Title/Abstract] OR "Alkaline phosphatase"[Title/Abstract] OR "Alkaline phosphatase"[MeSH Terms] OR "ALP"[Title/Abstract] OR "Gamma-Glutamyl transferase"[Title/Abstract] OR "GGT"[Title/Abstract] OR "lactate dehydrogenase"[Title/Abstract] OR "L-Lactate Dehydrogenase"[MeSH Terms] OR "L-Lactate Dehydrogenase"[Title/Abstract] OR "Dehydrogenase L-Lactate"[Title/Abstract] OR "Dehydrogenase Lactate"[Title/Abstract] OR "LDH"[Title/Abstract] OR "ast to alt ratio"[Title/Abstract] OR "ast to alt ratio"[Title/Abstract] OR "liver enzyme abnormality"[Title/Abstract] OR "liver enzyme activity"[Title/Abstract] OR "liver function tests"[MeSH Terms] OR "liver function tests"[Title/Abstract] OR "LEA"[Title/Abstract] OR "AST/ALT"[Title/Abstract]) AND ("Randomized Controlled Trial"[Title/Abstract] OR "clinical trial"[Title/Abstract] OR "controlled trial"[Title/Abstract] OR "intervention"[Title/Abstract] OR "Randomised"[Title/Abstract] OR "Randomized"[Title/Abstract] OR "randomly"[Title/Abstract] OR "placebo"[Title/Abstract] OR "trial"[Title/Abstract] OR "assignment"[Title/Abstract] OR "RCT"[Title/Abstract] OR "cross-over"[Title/Abstract] OR "parallel"[Title/Abstract] OR "single-blind"[Title/Abstract] OR "double-blind"[Title/Abstract] OR "Controlled Clinical Trial"[Title/Abstract]) |
| **Google scholar** | (“DASH” OR “dietary approaches to stop hypertension”) AND (“Liver” OR “liver enzyme” OR “Transaminases” OR “Aminotransferase” OR “transpeptidase” OR “Alanine Transaminase” OR “Alanine Aminotransferase” OR “ALT” OR “SGPT” OR “Aspartate Aminotransferases” OR “AST” OR “SGOT” OR “Alkaline phosphatase” OR “ALP” OR “Gamma-Glutamyl transferase” OR “GGT” OR “lactate dehydrogenase” OR “L-Lactate Dehydrogenase” OR “Dehydrogenase L-Lactate” OR “Dehydrogenase Lactate” OR “LDH” OR “AST-to-ALT ratio” OR “AST to ALT ratio” OR “liver enzyme abnormality” OR “liver enzyme activity” OR “liver function tests” OR “LEA” OR “AST/ALT”) AND ("Randomized Controlled Trial" OR "clinical trial" OR "controlled trial" OR "intervention" OR "Randomised" OR "Randomized" OR "randomly" OR "placebo" OR "trial" OR "assignment" OR "RCT" OR "cross-over" OR "parallel" OR "single-blind" OR "double-blind" OR "Controlled Clinical Trial") |
| **Web of science (68)** | #1 “DASH” (Topic) or “dietary approaches to stop hypertension” (Topic)  #2 “Liver” (Topic) or “liver enzyme” (Topic) or “Transaminases” (Topic) or “Aminotransferase” (Topic) or “transpeptidase” (Topic) or “Alanine Transaminase” (Topic) or “Alanine Aminotransferase” (Topic) or “ALT” (Topic) or “SGPT” (Topic) or “Aspartate Aminotransferases” (Topic) or “AST” (Topic) or “SGOT” (Topic) or “Alkaline phosphatase” (Topic) or “ALP” (Topic) or “Gamma-Glutamyl transferase” (Topic) or “GGT” (Topic) or “lactate dehydrogenase” (Topic) or “L-Lactate Dehydrogenase” (Topic) or “Dehydrogenase L-Lactate” (Topic) or “Dehydrogenase Lactate” (Topic) or “LDH” (Topic) or “AST-to-ALT ratio” (Topic) or “AST to ALT ratio” (Topic) or “liver enzyme abnormality” (Topic) or “liver enzyme activity” (Topic) or “liver function tests” (Topic) or “LEA” (Topic) or “AST/ALT” (Topic)  #3 "Randomized Controlled Trial" (Topic) or "clinical trial" (Topic) or "controlled trial" (Topic) or "intervention" (Topic) or "Randomised" (Topic) or "Randomized" (Topic) or "randomly" (Topic) or "placebo" (Topic) or "trial" (Topic) or "assignment" (Topic) or "RCT" (Topic) or "cross-over" (Topic) or "parallel" (Topic) or "single-blind" (Topic) or "double-blind" (Topic) or "Controlled Clinical Trial" (Topic)  #4 = #1 AND #2 AND #3 |

**Appendix S2.** Table of excluded studies at the full-text level, along with the reasons for their exclusion

| Studies did not report the relevant endpoints | N= 6 |
| --- | --- |
| Studies enrolled children or adolescents | N= 3 |
| Manuscripts were review articles or editorials | N= 5 |
